# Complementation of an *aglB* Mutant of *Methanococcus maripaludis* with Heterologous Oligosaccharyltransferases

**DOI:** 10.1371/journal.pone.0167611

**Published:** 2016-12-01

**Authors:** Yan Ding, Helen A. Vrionis, James Schneider, Alison Berezuk, Cezar M. Khursigara, Ken F. Jarrell

**Affiliations:** 1 Department of Biomedical and Molecular Sciences, Queen’s University, Kingston, Ontario, Canada; 2 Department of Molecular and Cellular Biology, University of Guelph, Guelph, Ontario, Canada; Tulane University Health Sciences Center, UNITED STATES

## Abstract

The oligosaccharyltransferase is the signature enzyme for *N*-linked glycosylation in all domains of life. In Archaea, this enzyme termed AglB, is responsible for transferring lipid carrier-linked glycans to select asparagine residues in a variety of target proteins including archaellins, S-layer proteins and pilins. This study investigated the ability of a variety of AglBs to compensate for the oligosaccharyltransferase activity in *Methanococcus maripaludis* deleted for *aglB*, using archaellin FlaB2 as the reporter protein since all archaellins in *Mc*. *maripaludis* are modified at multiple sites by an *N*-linked tetrasaccharide and this modification is required for archaellation. In the *Mc*. *maripaludis* Δ*aglB* strain FlaB2 runs as at a smaller apparent molecular weight in western blots and is nonarchaellated. We demonstrate that AglBs from *Methanococcus voltae* and *Methanothermococcus thermolithotrophicus* functionally replaced the oligosaccharyltransferase activity missing in the *Mc*. *maripaludis* Δ*aglB* strain, both returning the apparent molecular weight of FlaB2 to wildtype size and restoring archaellation. This demonstrates that AglB from *Mc*. *voltae* has a relaxed specificity for the linking sugar of the transferred glycan since while the *N*-linked glycan present in *Mc*. *voltae* is similar to that of *Mc*. *maripaludis*, the *Mc*. *voltae* glycan uses *N*-acetylglucosamine as the linking sugar. In *Mc*. *maripaludis* that role is held by *N*-acetylgalactosamine. This study also identifies *aglB* from *Mtc*. *thermolithotrophicus* for the first time by its activity. Attempts to use AglB from *Methanocaldococcus jannaschii*, *Haloferax volcanii* or *Sulfolobus acidocaldarius* to functionally replace the oligosaccharyltransferase activity missing in the *Mc*. *maripaludis* Δ*aglB* strain were unsuccessful.

## Introduction

*N*-glycosylation refers to the covalent attachment of glycans to target proteins at asparagine residues located within a conserved sequon (Asn-X-Ser/Thr, where X can be any amino acid except proline). The oligosaccharyltransferase (OST) is the signature enzyme of the *N*-glycosylation pathways in all three domains of life [1–3]. In both prokaryotic domains, Bacteria and Archaea, the OST exists as a single subunit designated PglB [4] and AglB [5], respectively. In higher Eukaryotes, the OST is a multi-subunit complex with the Stt3 subunit identified as the catalytic subunit [6]. Both PglB and AglB are homologs of Stt3 [1,4].

The *N*-glycosylation pathway in Archaea has been best studied, in terms of both glycan structure and genetic analysis of the biosynthesis and assembly of the glycan, in three distinctive model organisms, namely the methanogen *Methanococcus maripaludis*, the extreme halophile *Haloferax volcanii* and the thermoacidophile *Sulfolobus acidocaldarius* [1,7,8]. Glycan structures alone are published for several other varied members of this domain [1,9,11]. The most commonly studied proteins modified by *N*-linked glycans in archaea are S-layer proteins [12–14], archaellins [15–19] and pilins [20–22]. The current model of the archaeal *N*-glycosylation pathway involves the sequential addition of sugar monomers onto a dolichol-type lipid carrier embedded in the cytoplasmic membrane, followed by a flipping of the lipid-linked glycan across the membrane. Finally, on the external face side of the cytoplasmic membrane, AglB transfers the glycan from the lipid carrier onto the acceptor protein *en bloc* [1,23]. Further addition of sugar monomers onto the protein-bound *N*-glycan has been shown to occur in a very limited number of archaeal species [24,25].

Among the three domains, it appears that glycan structures as well as the nature of the linking sugar and the lipid carrier are most variable in Archaea [23]. In different archaea, the sugar linking the glycan to the asparagine of the target protein can be *N*-acetyl-glucosamine, *N*-acetyl-galactosamine or a simple hexose [1]. The lipid carrier has been identified in several archaea and can be dolichol phosphate or dolichol diphosphate and the dolichol can vary in length as well as degree of saturation [2,24,26–30]. These are all aspects that may affect AglB activity. In both *Mc*. *maripaludis* and *Hfx*. *volcanii*, *aglB* deletion mutants have been isolated [5,12,31] while the enzyme is essential for *S*. *acidocaldarius* [32].

The specificity of AglB for different sugar-donor substrates was first reported for enzymes from *Archeoglobus fulgidus* and *Pyrococcus furiosus*. In *in vitro* experiments, neither enzyme could process the lipid-linked glycan of the other organism [33], indicating specificity of the enzyme but it is unclear whether this is due to the structure of the different glycans or other factors. More recently, Eichler’s group showed substrate promiscuity of AglB from various extreme halophiles [34]. Specifically, AglB from *Haloarcula marismortui*, *Halobacterium salinarum* and *Haloferax mediterranei* could all functionally replace the oligosaccharyltransferase activity in a *Hfx*. *volcanii* Δ*aglB* strain. While all of these species are extreme halophiles, their respective AglBs, though structurally similar, all transfer lipid-linked glycans in their native cells that are distinct from that found in *Hfx*. *volcanii*. In addition, in at least some cases, the heterologous AglB had to accommodate a different linking sugar or dolichol length to be effective, demonstrating a relaxed specificity of the enzymes.

The overwhelming majority of archaea (166/168 sequenced genomes examined) contain an identifiable *aglB* [35], including a significant number of organisms (>30) that appear to possess multiple copies of the gene. Examination of the multiple copies of *aglB* within a given organism indicated that, at least in some cases, distinct versions of the enzyme are present that possess, for example, variations in the catalytic motif WWDXG. Other studies have indicated that not all versions of AglB are constitutively expressed in organisms that have multiple copies [10,24]. In the case of *Hfx*. *volcanii*, two distinct *N*-linked glycans have been reported, depending on the salt concentration of the medium [36]. The transfer of the low salinity glycan still occurs in an *aglB* mutant even though only a single *aglB* gene is detected in the sequenced genome [35] suggesting the existence of either an additional *aglB* with such low sequence similarity to known *aglBs* that it escaped detection or a novel mechanism/enzyme for the transfer of the low salinity glycan. The presence of multiple AglBs in a single organism, some not constitutively expressed, some with variations in conserved motifs, suggest that they may be involved in different transfer reactions with different substrates. Such is the case in *Trypanosoma brucei*, where there are three single subunit OSTases, all with distinct donor and acceptor specificities [37].

In *Mc*. *maripaludis*, *N*-glycosylation of archaellins (the structural proteins of the motility apparatus archaella [38,39]) is critical for their assembly into archaella [31,40,41]. In wildtype cells, the archaellins are decorated at multiple locations with a tetrasaccharide [17]. In mutants in which the resulting glycan is truncated, the shortened glycan, even a monosaccharide version, is still efficiently transferred to the target archaellins indicating that AglB does not require the complete glycan structure for transfer [31]. *In vitro* assays using purified *Mc*. *voltae* AglB showed efficient transfer of a truncated disaccharide glycan but not if Dol-P with a monosaccharide was used as the donor [2]. While cells can assemble functional archaella with only a truncated disaccharide attached to archaellins, if a mutation occurs that results in the glycan being truncated to a single sugar or in archaellins that are completely non-glycosylated, then the cells are nonarchaellated [31]. The latter case occurs if *aglB* is deleted. Interestingly, in cases where genes are deleted that result in nonarchaellated cells, such as the *ΔaglB* deletion mutant, these mutants subsequently stop transcription of the *fla* operon, a series of genes which includes the three archaellin genes as well as a number of accessory genes (*flaC-J*) also required for archaella assembly [42]. This occurs, at least in some cases, due to a second mutation in a recently described transcriptional activator for the *fla* operon, EarA [43].

In this contribution, we examine the ability of several heterologous AglBs to functionally compensate for the oligosaccharyltransferase activity in a *Mc*. *maripaludis* strain deleted for *aglB*. This information contributes to the substrate specificity/variability of AglB and may aid in studies designed to use Archaea for glyco-engineering purposes [44,45].

## Materials and Methods

### Strains and growth conditions

*Methanococcus maripaludis* S2 *Δhpt* (Mm900) [46], *Methanococcus voltae* PS, *Methanothermococcus thermolithotrophicus* DSM2095 were all grown in Balch Medium III [47] under a headspace of CO_2_/H_2_ (20:80). *Methanocaldococcus jannaschii* JAL-1 was grown in the minimal medium described by Ferrante et al. [48]. *Mc*. *maripaludis* and *Mc*. *voltae* were incubated at 35°C, *Mtc*. *thermolithotrophicus* at 60°C and *Mcc*. *jannaschii* at 80°C. For complementation studies, *Mc*. *maripaludis* Δ*aglB* harbouring the various complementation vectors were grown in nitrogen-free medium containing puromycin (2.5μg/ml) for plasmid selection and supplemented with either L-alanine (10mM) or NH_4_Cl (10mM) as sole nitrogen source [49]. *Escherichia coli* Top10 cells (Invitrogen, Burlington ON, Canada), used for various cloning steps, were grown at 37°C in Luria-Bertani (LB) broth or agar with ampicillin added (100μg/ml) for plasmid selection when required.

### Isolation of a *Mc*. *maripaludis* Δ*aglB* mutant

An in-frame deletion of *aglB* in *Mc*. *maripaludis* was re-created using pKJ574 and methodology previously reported [31]. Following confirmation of the inframe deletion by PCR screening and sequencing this strain was designated as Δ*aglB*-14-9.

### Cloning of various *aglBs*

*aglB*, from *Mc*. *voltae* (GenBank accession ABD17750), was amplified by PCR (50°C annealing temperature, 3 min extension, 30 cycles) using primers listed in Table 1 and washed *Mc*. *voltae* cells as template. The forward and reverse primers had either NsiI or MluI restriction sites added, respectively. For *Mtc*. *thermolithotrophicus*, the *aglB* sequence has not been previously reported. A BLAST search using the AglB protein sequence from *Mc*. *maripaludis* as bait, retrieved WP_018154595 in GenPept as the only hit (100% coverage, 59% identity, 77% similarity) in *Mtc*. *thermolithotrophicus* DSM 2095. The gene (GenBank accession NZ_AQXV01000054.1) was amplified by PCR (50°C annealing temperature, 3 min extension, 30 cycles) using the primers listed in Table 1 and washed cells of *M*. *thermolithotrophicus* as template. The *aglB* gene from *Mcc*. *jannaschii* (MJ_RS08150) was amplified by PCR (50°C annealing temperature, 3 min extension, 30 cycles) using the primers listed in Table 1 and washed cells of *Mcc*. *jannaschii* as template. The forward primer in this case included a single nucleotide change to remove an internal NsiI restriction site while leaving the amino acid sequence unchanged. The *aglB* gene from *Hfx*. *volcanii* (HVO_RS12050) was amplified by PCR (62.5°C annealing temperature, 3 min extension, 30 cycles) with the primers listed in Table 1 and genomic DNA (gift of Jerry Eichler) as template. *Hfx*. *volcanii aglB* and *Sulfolobus acidocaldarius aglB* (NC_007181.1) were also synthesized with a C-terminal FLAG-tag using *Mc*. *maripaludis* codon preferences while avoiding NsiI and MluI sites (GenScript, NJ). Each of the *aglB* genes was digested with NsiI and MluI and cloned into pHW40 previously digested with the same restriction enzymes to generate the complementation plasmids used in this study (Table 2). Transcription of the cloned *aglB* is under the control of a regulatable *nif* promoter in this vector. The complementation plasmid carrying the *Mc*. *maripaludis aglB*, pKJ677, was previously constructed [31]. A site directed mutagenesis step to remove an internal NsiI restriction site was performed prior to the cloning of the *Mc*. *maripaludis aglB* into pHW40 as an NsiI/XbaI fragment (see Table 1 for primer pair used).

**Table 1:** Primers used in this study.

Primers	Sequence (5’ to 3’)	Restriction site (underlined)
**AglB complementation**		
MaraglB-F	CCCATGCATGGGTGAATTTTTAAATAAAGTC	NsiI
MaraglB-R	GCTCTAGATTAGTGATGGTGGTGATGATGATTGAGATAGTCAGTTCCA	XbaI
VoltaglB-F	CGTAATGCATGACTGAAAACAACGAAAAAGTCAAAAATTCCGATTCTGC	NsiI
VoltaglB-R	GACTACGCGTTTATTTTGAGTAATTACCGTAATCCACGC	MluI
ThermaglB-F	CCAATGCATGGGGGAATTCCTGAACAAATTTTCC	NsiI
ThermaglB-R	AGCACGCGTTTAGTTAAGATAATCTATTCCATAATC	MluI
JannaglB-F*	CCAATGCATGTATATAAAGGTGAAACTTATGAGTAATGC**T**TTAG	NsiI
JannaglB-R	AGCACGCGTTATTTTAGATAATCAGTTCCATAATC	MluI
HvaglB-F	CAGTATGCATGAGTGACGAGCAAACAAAGTATTCG	NsiI
HvaglB-R	TACGTCTAGATTACGCGGCGGCCGAGACCG	XbaI
SDM**		
MaraglBSDM-F	GAAGTTGAAAACGCATGCCCAACC	
MaraglBSDM-R	GGTTGGGCATGCGTTTTCAACTTC	
**RT-PCR**		
SaaglB-RT-F	CACACTTACTCTTGTTTCACC	
SaaglB-RT-R	GCATCTCGAGCCAGTTATCGGCAGAATCGTAG	
HvaglB-RT-F	GATTCGCAGACCACAACATCG	
HvaglB-RT-R	GAAGTTTCTTGCGATTGAGTCG	

*T shown in bold underline removes and internal *NsiI* site

**SDM primers remove internal *NsiI* site in *Mc*. *maripaludis aglB*. Change is underlined

**Table 2:** Plasmids used in this study.

Plasmids	Description	Reference/Source
pHW40	*nif* promoter-*lacZ* fusion plus Pur^r^ cassette;Amp^r^	John Leigh
pCRPrtNeo	*hmv* promoter-*hpt* fusion plus Neo^r^ cassette in pCR2.1Topo; Amp^r^	[46]
pKJ574	pCRPrtNeo harboring an inframe deletion of *aglB*	[31]
pKJ677	pHW40 harboring *Mc*. *maripaludis aglB*	This study
pKJ1229	pHW40 harboring *Mc*. *voltae aglB*	This study
pKJ1240*	pHW40 harboring *S*. *acidocaldarius aglB*	This study
pKJ1248	pHW40 harboring *Mtc*. *thermolithotrophicus aglB*	This study
pKJ1251*	pHW40 harboring *Hfx*. *volcanii aglB*	This study
pKJ1267	pHW40 harboring *Mcc*. *jannaschii aglB*	This study

*gene synthesized using *Mc*. *maripaludis* codon preferences

### Complementation of the *Mc*. *maripaludis* Δ*aglB* mutant

*Mc*. *maripaludis* Δ*aglB* 14–9 was transformed with the various pHW40-derived plasmids containing different *aglB* genes using the PEG precipitation method [50]. The complementation strains were subsequently grown in the presence of puromycin (2.5 μg/ml) in nitrogen-free medium supplemented with either 10 mM NH_4_Cl (*nif* promoter repressed) or alanine (*nif* promoter induced). At least three transfers in nitrogen-free medium supplemented with alanine were done prior to analysis.

### Reverse transcription polymerase chain reaction (RT-PCR)

Total RNA from *ΔaglB* mutant cells carrying either pKJ1240 (with the *Mc*. *maripaludis* codon-optimized *aglB* from *S*. *acidocaldarius*, *aglB*_*sa*_) or pKJ1251 (with the *Mc*. *maripaludis* codon-optimized *aglB* from *Hfx*. *volcanii*, *aglB*_*hv*_) was extracted using a High Pure RNA Isolation Kit (Roche Life Science), followed with an additional DNase digest using an Ambion^™^ TURBO DNA-free Kit (Invitrogen). The presence of the *aglB*_*sa*_ or *aglB*_*hv*_ transcript in the RNA extract was detected using a OneStep RT-PCR Kit (Qiagen) with 10 ng of the total RNA extract as template and the corresponding gene-specific primers listed in Table 1. After a 30 min reverse transcription step at 50°C and a 15 min initial PCR activation step at 94°C, the polymerase chain reaction consisted of 30 cycles of 30 sec denaturation at 94°C, 30 sec annealing at 48°C, and 30 sec extension at 72°C. Amplification experiments using RNA extracts from *ΔaglB* mutant cells carrying either pKJ1240 or pKJ1251 as template and the corresponding gene-specific primer pair, but omitting the reverse transcription step were conducted to rule out the possibility of DNA contamination of the RNA template.

PCR experiments using pKJ1240 or pKJ1251 as template and the corresponding gene-specific primer pairs were performed to confirm the amplicon size and primer specificity in the RT-PCR. In addition, RT-PCR experiments were also conducted using RNA extract from *ΔaglB* mutant cells carrying plasmid-borne *aglB*_*sa*_ (pKJ1240) as template and the *aglB*_*hv*_ primer pair and RNA extract from *ΔaglB* mutant cells carrying plasmid-borne *algB*_*hv* (_pKJ1251) as template and the *aglB*_*sa*_ primer pair to further exclude the possibility of non-specific amplification of the two primer pairs.

### Western blotting

Whole cell lysates of *Mc*. *maripaludis* Mm900, *Mc*. *maripaludis* Δ*aglB*-14-9 and the various *Mc*. *maripaludis* Δ*aglB-14-9* strains carrying complementation plasmids were separated by SDS-PAGE (12.5% acrylamide gels) and transferred to Immobilon-P membrane (Millipore, MA) and examined by western blotting using anti-FlaB2 specific antibodies, as previously described [42]. Complemented strains were transferred three times in nitrogen-free medium supplemented with either NH_4_Cl or alanine prior to the western blotting experiments. Detection of FLAG-tagged versions of *Hfx*. *volcanii* and *S*. *acidocaldarius* AglB in whole cell lysates of the appropriate complementation strains was attempted using anti-FLAG antibodies (mouse monoclonal, Sigma).

### Electron microscopy

Transmission electron microscopy was performed on the various *Mc*. *maripaludis* strains after staining with 2% (w/v) phosphotungstic acid, pH 7.0, as previously described [43].

### Bioinformatics

The presence of transmembrane domains in AglB proteins selected for this study was predicted using HMMTOP (http://www.enzim.hu/hmmtop/) [51].

## Results and Discussion

### AglB from *Mc*. *maripaludis* restores *N*-glycan modification of FlaB2 and archaellation in the Δ*aglB*-14-9 mutant

AglB is the oligosaccharyltransferase that performs the most conserved and critical terminal step in the *N*-glycosylation pathway in Archaea, namely the transfer of a glycan from its lipid carrier to the target protein [5]. Following the initial isolation of the first *aglB* deletion strain in *Mc*. *maripaludis*, attempts were made to complement that strain with a wildtype copy of *aglB* expressed *in trans*. However, it was not possible to confirm that the expressed wildtype copy of *aglB* could rescue the deletion strain since the *aglB* mutant quickly stopped synthesis of FlaB2 upon repeated serial transfers [31]. This turned out to be a common occurrence for any mutant carrying a deletion in any gene that resulted in nonarchaellated cells [43].

**Fig 1:**
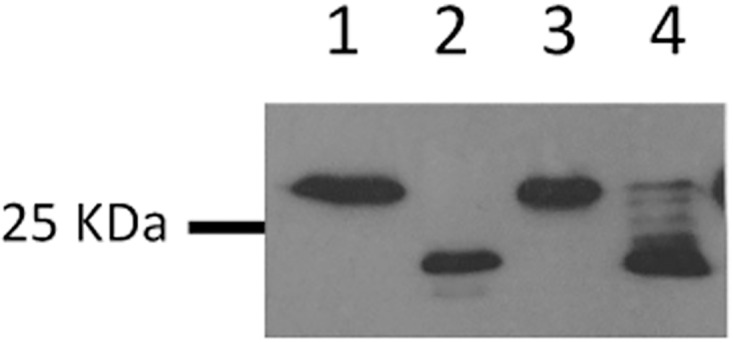
Western blot analysis of FlaB2 in an *Mc*. *maripaludis* Δ*aglB*-*14-9* mutant complemented *in trans* with homologous *aglB*. The Δ*aglB-14-9* mutant was complemented with a plasmid borne version of *Mc*. *maripaludis aglB* under the control of the *nif* promoter. The complemented Δ*aglB-14-9* mutant was grown in nitrogen-free medium supplemented with alanine or NH_4_Cl which results in transcription from the *nif* promoter being on (alanine) or off (NH_4_Cl). Lane 1 is Mm900 (WT), lane 2 is Δ*aglB-14-9* mutant, lane 3 is Δ*aglB-14-9* mutant complemented cells grown in nitrogen-free medium supplemented with alanine, lane 4 is Δ*aglB-14-9* mutant complemented cells grown in nitrogen-free medium supplemented with NH_4_Cl.

**Fig 2:**
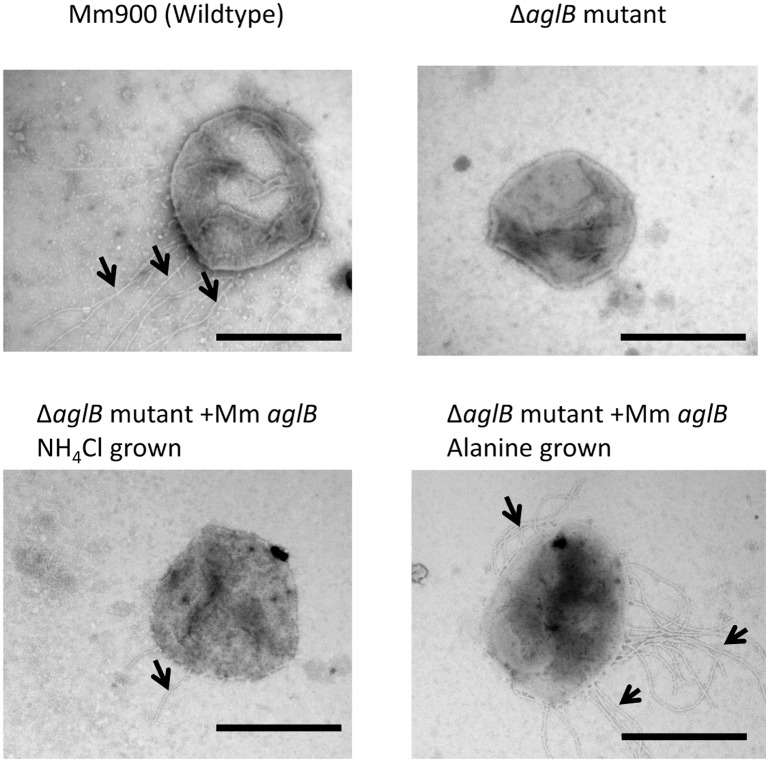
Electron micrographs of the *Mc*. *maripaludis* Δ*aglB-14-9* mutant complemented *in trans* with homologous *aglB*. The Δ*aglB-14-9* mutant was complemented with a plasmid borne versions of *Mc*. *maripaludis aglB* under the control of the *nif* promoter. Complemented cells were examined after a minimum of three transfers in nitrogen-free medium supplemented with NH_4_Cl or alanine by negative staining (2% phosphotungstic acid, pH 7.0). Arrows indicate archaella. Bar equals 1μm.

Thus, for this study, we initially had to recreate the *aglB* deletion and immediately store aliquots of the mutant, designated Δ*aglB-14-9*, at -80°C while it was still synthesizing FlaB2 that could be detected in western blots. Using pKJ677 expressing wildtype *Mc*. *maripaludis aglB* from a *nif* promoter, it was possible to show complementation of the deleted *aglB* in the *ΔaglB-14-9* mutant. This was initially demonstrated by western blotting where the faster migrating version of the reporter glycoprotein FlaB2 in *ΔaglB-14-9* was fully restored to wildtype size following growth of the complemented strain in nitrogen-free medium supplemented with alanine, where transcription from the *nif* promoter is induced (Fig 1). A small amount of the wildtype size FlaB2, in addition to minor amounts of intermediate sized FlaB2, was also observed when complemented cells were grown in nitrogen-free medium supplemented with NH_4_Cl, conditions in which transcription from the *nif* promoter is repressed. This has been observed previously in complementation experiments and attributed to very small amounts of transcription from the *nif* promoter even under NH_4_Cl growth conditions [31,40,52,53]. *aglB* deletion mutants are nonarchaellated due to a requirement for at least a truncated disaccharide version of the wildtype tetrasaccharide *N*-linked glycan to be attached to the archaellins for their assembly into archaella [31]. Thus, complemented cells were also examined by electron microscopy for the presence of archaella. Such examination revealed that under alanine growth conditions the complemented *ΔaglB-14-9* cells were now archaellated (Fig 2).

### Comparison of selected AglBs used in this study

To study the promiscuous nature of archaeal AglBs, the *ΔaglB-14-9* strain was also complemented with various heterologous *aglBs*, including from other methanogens (*Mc*. *voltae*, *Mtc*. *thermolithotrophicus* and *Mcc*. *jannaschii*) and from other more distantly related archaeal species in which the *N*-glycosylation system has been best studied, namely *S*. *acidocaldarius* and *Hfx*. *volcanii*. Successful complementation would also indicate, in cases where it was not yet known (*Mtc*. *thermolithotrophicus* and *Mcc*. *jannaschii*), that the putative *aglB* gene did encode an active oligosaccharyltransferase. The topology of AglB proteins include a typical 13 transmembrane helices in the N-termini with extracellular loops between transmembrane helixes, and a soluble C-terminal domain located in the extracellular side of the cytoplasmic membrane where the catalytic site is located [54–56]. A pairwise comparison of the *Mc*. *maripaludis* AglB to the other AglBs used in this study is presented in Table 3 (see also S1–S5 Figs for EMBOSS Needle pairwise alignments). The three methanogen AglBs used in this study were from the mesophilic *Mc*. *voltae*, the thermophilic *Mtc*. *thermolithotrophicus* (optimal growth at 60–65°C) and the hyperthermophilic *Mcc*. *jannaschii* (optimal growth at 80–85°C). These methanogens, as well as *Mc*. *maripaludis*, are all stringent anaerobes that grow optimally at neutral pH with 1–4% NaCl. *Hfx*. *volcanii* is an extreme halophile growing optimally in 1.5–2.5M NaCl, at pH 7 and 45°C. *S*. *acidocaldarius*, on the other hand, is a thermoacidophile, growing optimally at 70–75°C and pH 2–3. Since the catalytic site in AglB is predicted to be orientated extracellularly [34], the enzymes from *Hfx*. *volcanii* and *S*. *acidocaldarius* may have specific adaptations to function in high salt or low pH that would not be required for the methanogen AglBs.

**Table 3:** Comparison of *Mc*. *maripaludis* AglB to other AglBs used in this study (pairwise EMBOSS Needle).

Organism	Length (aa)	TMDs	% identity	% similarity	Gaps
*Mc*. *maripaludis*	852	13			
*Mc*. *voltae*	917	13	49.2	66.3	8.8
*Mtc*. *thermolithotrophicus*	860	13	59.4	77.5	1.4
*Mcc*. *jannaschii*	933	15	46.9	62.6	13.7
*Hfx*. *volcanii*	1054	14	17.9	31.0	39.7
*S*. *acidocaldarius*	754	15	19.9	34.6	30.1

AglB of *Mtc*. *thermolithotrophicus* was most similar to AglB of *Mc*. *maripaludis*, being almost 60% identical (and 77.5% similar) and almost identical in length (860 amino acids vs 852 for *Mc*. *maripaludis* AglB). AglBs from *Mc*. *voltae* and *Mcc*. *jannaschii* were larger by 65 and 81 amino acids, respectively and slightly less than 50% identical to the *Mc*. *maripaludis* enzyme. Most of the extra length in the *Mcc*. *jannaschii* enzyme is found in one large insertion of about 70 amino acids (between amino acids 227 and 299), when compared to the *Mc*. *maripaludis* AglB. This insertion is predicted to contain 2 additional transmembrane helices, resulting in a total of 15, compared to 13 for AglBs of *Mc*. *maripaludis*, *Mc*. *voltae* and *Mtc*. *thermothithotrophicus*. The AglBs from the two non-methanogens tested, *Hfx*. *volcanii* and *S*. *acidocaldarius*, were much less similar to the *Mc*. *maripaludis* AglB, being only 17.9 and 19.9% identical, respectively. In addition, these two oligosaccharyltransferases were much different in length compared to the *Mc*. *maripaludis* AglB. The *Hfx*. *volcanii* AglB was almost 200 amino acids larger than the *Mc*. *maripaludis* enzyme while that of *S*. *acidocaldarius* was almost 100 amino acids smaller.

**Fig 3:**
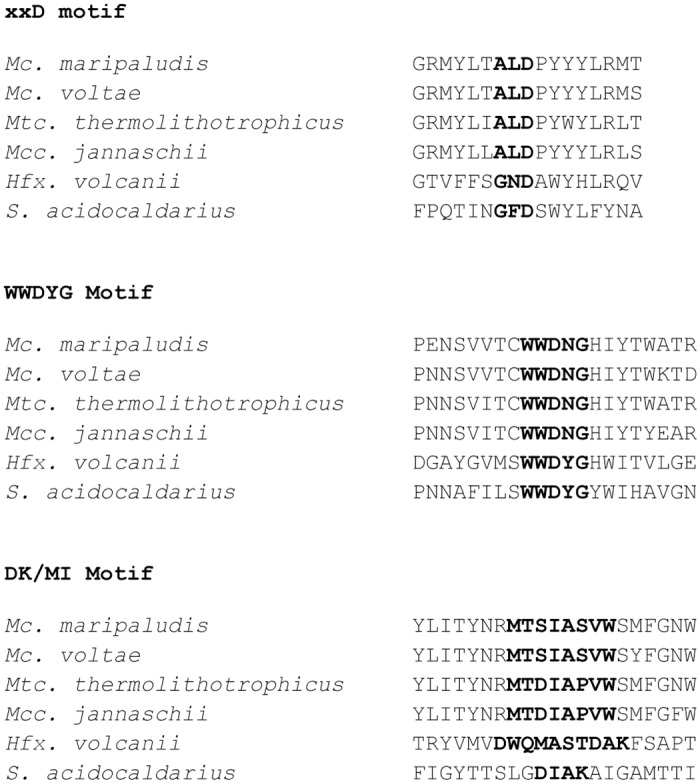
Comparison of the conserved motifs in the various AglBs used in this study. The putative xxD, WWDXG and DK/MI motifs for the 6 AglBs used in this study are indicated in bold.

Examination of the 6 AglBs revealed differences in conserved motifs previously shown to be important for catalysis (Fig 3). The xxD motif (sometimes referred to as the ExD or DxD motif) is thought to bind the dolichol carrier (DolP or DolPP). In all four methanogen AglBs, this motif is ALD, while in *Hfx*. *volcanii* it is GND and in *S*. *acidocaldarius* it is GFD. In all cases, this motif is found in the first extracellular loop in the N-terminus of the protein. The second motif, WWDXG, is necessary for catalysis and found in the extracellular C-terminal domain. In the four methanogens, this motif is identical, WWDNG, while in both *Hfx*. *volcanii* and *S*. *acidocaldarius* the sequence of this motif is WWDYG. Most variation is found in the third motif, the DK (**D**XX**K** or the relaxed version **D**XX**M**XXX(**M/I**)) or MI (**M**XX**I**XXX(**I/V/W**) motif [57]. This motif helps to form the pocket that recognizes the serine or threonine residue found in the *N*-linked glycosylation sequon (N-X-S/T) and is found adjacent to the WWDXG motif in the tertiary structure of the enzymes. The DK motif is found in most AglB proteins (as well as in eukaryotic Stt3) although variations in the motif have been reported in terms of an insertion of 4–14 amino acids that interrupts the DK motif in some archaea (**D**XX**M**X(4–14)**K**), including in certain extreme halophiles. *Hfx*. *volcanii* AglB has a 5 amino acid insertion here. *S*. *acidocaldarius* AglB has the DK motif (**D**IA**K**) which is typical of Crenarchaeota. In lieu of the DK motif, some archaea (as well as bacterial PglBs) have an MI motif at the same position which is thought to perform the same function as the DK motif. This is the case for the four methanogens in this study. The three key amino acids are conserved in all four cases (**M**XX**I**XXX**W**) although there is some variation in the X positions (Fig 3). Since the side chains of the amino acids that comprise the DK and MI motif are very different, it is unclear what differences in substrate recognition this variation may have [57].

### AglB homologues from *Mc*. *voltae* and *Mtc*. *thermolithotrophicus* but not *Mcc*. *jannaschii* could functionally replace the OST activity in *Mc*. *maripaludis* Δ*aglB-14-9*

**Fig 4:**
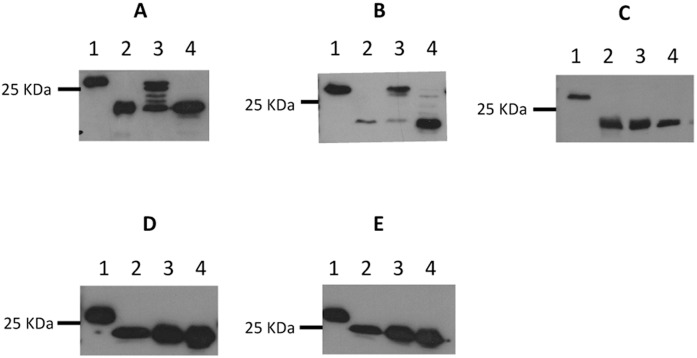
Western blot analysis of FlaB2 in an *Mc*. *maripaludis* Δ*aglB-14-9* mutant complemented *in trans* with heterologous *aglBs*. The Δ*aglB-14-9* mutant was complemented with plasmid borne versions of heterologous *aglB* from *Mc*. *voltae* (A), *Mtc*. *thermolithotrophicus* (B), *Mcc*. *jannaschii* (C), *Hfx*. *volcanii* (D) or S. *acidocaldarius* (E). For D and E the *aglB* was synthesized using *Mc*. *maripaludis* codon preferences. The complemented Δ*aglB-14-9* mutant was grown in nitrogen-free medium supplemented with alanine or NH_4_Cl which results in transcription from the *nif* promoter being on (alanine) or off (NH_4_Cl). In each panel, lane 1 is Mm900 (WT), lane 2 is Δ*aglB-14-9* mutant, lane 3 is Δ*aglB-14-9* mutant complemented cells grown in nitrogen-free medium supplemented with alanine, lane 4 is Δ*aglB-14-9* mutant complemented cells grown in nitrogen-free medium supplemented with NH_4_Cl.

**Fig 5:**
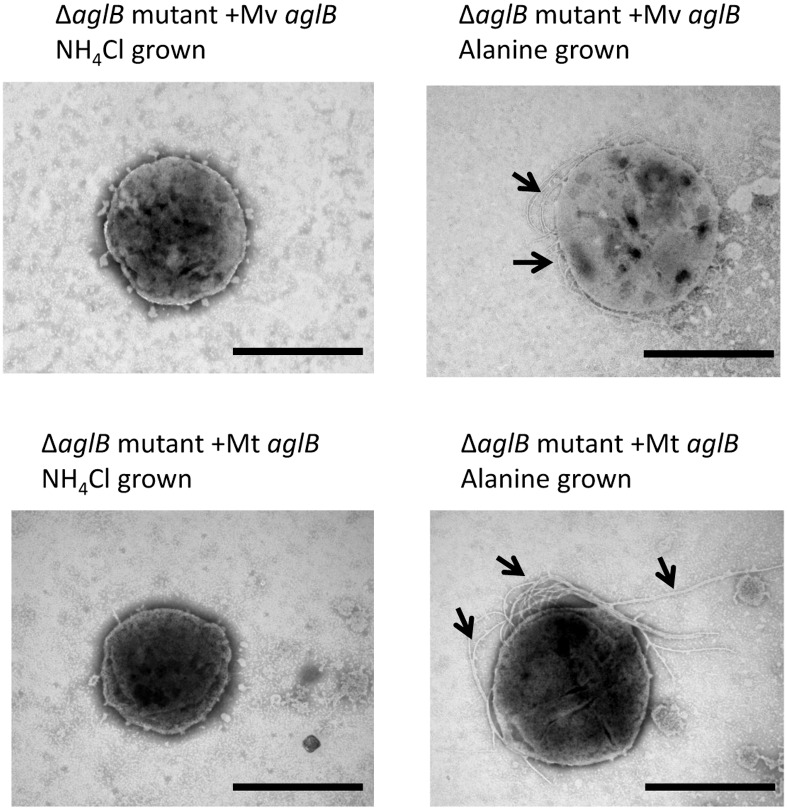
Electron micrographs of the *Mc*. *maripaludis* Δ*aglB-14-9* mutant complemented *in trans* with heterologous *aglBs*. The Δ*aglB-14-9* mutant was complemented with plasmid borne versions of *aglB* from *Mc*. *voltae* (Mv *aglB*) or *Mtc*. *thermolithotrophicus* (Mt *aglB*). Complemented cells were examined after a minimum of three transfers in nitrogen-free medium supplemented with NH_4_Cl or alanine by negative staining (2% phosphotungstic acid, pH 7.0). Arrows indicate archaella. Bar equals 1μm.

In all attempted cases of heterologous complementation of the *Mc*. *maripaludis* Δ*aglB* mutant, the initial screen to indicate successful complementation was by western blotting after growth of the complemented Δ*aglB-14-9* strain in nitrogen-free medium supplemented with alanine or NH_4_Cl. Only a limited number of archaeal *N*-linked glycan structures are known [1] and of the three other methanogens tested in this study only in *Mc*. *voltae* has an *N*-linked glycan structure been reported. It is a trisaccharide with significant similarity to the tetrasaccharide of *Mc*. *maripaludis* [15,17]. Interestingly, however, the linking sugar is different, though both are *N*-acetylated. In the case of *Mc*. *voltae*, this position is held by an *N*-acetyl-glucosamine while in *Mc*. *maripaludis* the linking sugar in the glycan is *N*-acetyl-galactosamine. Transformation of the Δ*aglB-14-9* strain with pKJ1229 containing *Mc*. *voltae aglB*, followed by growth in alanine supplemented nitrogen-free medium indicated that the *Mc*. *voltae* enzyme was capable of restoring the lost oligosaccharyltransferase activity leading to the production of wildtype sized FlaB2s (Fig 4A) which were assembled into archaella (Fig 5). Clearly, the *Mc*. *voltae* enzyme is active on glycan structures with either GlcNAc or GalNAc as the linking sugar. The promiscuity towards glycans with different linking sugars is similar to reports in the halophile system where AglBs from different halophiles can transfer glycans with hexose or *N*-acetylhexuronic acid acting as the linking sugar [34]. This confirms by enzyme activity the *aglB* gene of *Mc*. *voltae* previously identified only by deletion analysis [5].

*aglB* from two other genera of *Methanococcales* with higher optimal growth temperatures was also tested for their ability to complement the Δ*aglB-14-9* strain. For both *Mtc*. *thermolithotrophicus* and *Mcc*. *jannaschii*, no *N*–linked glycan structures have been reported, although the archaellins of *Mtc*. *thermolithotrophicus* contain many potential glycosylation sequons [58] and were reported to stain weakly with glycoprotein stains [59]. *Mtc*. *thermolithotrophicus* was not one of the 168 species of archaea examined for the presence of *aglB* by the Eichler group [35] since a complete, annotated genome is not available. However, a BLAST search revealed a single *Mtc*. *thermolithotrophicus* gene (GenBank accession No. NZ_AQXV01000054.1) from a whole genome shotgun sequence (GenBank accession No. NZ_AQXV01000019) with high sequence identity to *aglB* from *Mc*. *maripaludis*. Despite being a thermophile with optimal growth near 60°C, this gene from *Mtc*. *thermolithotrophicus* was able to very effectively restore wildtype size FlaB2 (Fig 4B) as well as archaellation (Fig 5) to the Δ*aglB-14-9* strain grown at 35°C when used in complementation studies, thereby confirming the identity of the *Mtc*. *thermolithotrophicus* OST by activity.

All attempts to show complementation of the Δ*aglB-14-9* strain with the *Mcc*. *jannaschii aglB* homolog were unsuccessful (Fig 4C). The failure of the *Mcc*. *jannaschii* AglB to complement is intriguing. This AglB has very high sequence identity and similarity to that of *Mc*. *maripaludis*. Furthermore, the three conserved motifs examined had identical sequences in the *Mcc*. *jannaschii* AglB to that found in AglB from *Mtc*. *thermolithotrophicus*, which did complement the *aglB* deletion strain of *Mc*. *maripaludis*. Strikingly though, there is a large insertion (approximately 70 amino acids) found in the *Mcc*. *jannaschii* AglB lacking in the remaining three methanogen AglBs. The lack of complementation may be due simply to the activity profile of the *Mcc*. *jannaschii* AglB in regards to temperature. Complementations of the *aglB* mutant of *Mc*. *maripaludis* were done at 35°C and 40°C while *Mcc*. *jannaschii* grows optimally above 80°C. Growth of *Mc*. *maripaludis* can occur to 45°C but above 42°C the reporter protein we use, FlaB2 archaellin, is not made [60]. However, other possible reasons for the failure of this complementation can also be considered. While all *aglB*s were cloned into the same site of the same vector and transcribed from the same *nif* promoter, we cannot rule out that the lack of activity demonstrated was due to degradation/instability of the expressed *Mcc*. *jannaschii* AglB. Furthermore, the lipid composition of the cytoplasmic membrane of *Mcc*. *jannaschii* is known to vary considerably depending on the growth temperature. At the low end of its growth range (45°C), the lipids are mostly typical diethers, but as the growth temperature is raised to 75°C the proportion of tetraether lipids and a novel macrocyclic diether lipid dramatically increase [61]. The inability of the *Mcc*. *jannaschii aglB* to complement in *Mc*. *maripaludis* may thus be due to faulty insertion of the AglB into the strictly diether lipids in the cytoplasmic membrane of *Mc*. *maripaludis* [62]. This would not have been a problem for the AglBs of *Mc*. *voltae* or *Mtc*. *thermolithotrophicus* since both these organisms have membrane lipids that are also diethers [63]. As with *Mtc*. *thermolithotrophicus*, no *N*-linked glycan structures have been reported for *Mcc*. *jannaschii* and, likewise, there are no reports on the structures of dolichol phosphate carriers so it is possible that AglB in *Mcc*. *jannaschii* would naturally recognize a unique glycan structure possibly with a different linking sugar or a different lipid carrier and was not able to utilize the *Mc*. *maripaludis* versions.

### AglB homologues from *S*. *acidocaldarius and Hfx*. *volcanii* could not functionally replace the OST activity in *Mc*. *maripaludis* Δ*aglB-14-9*

**Fig 6:**
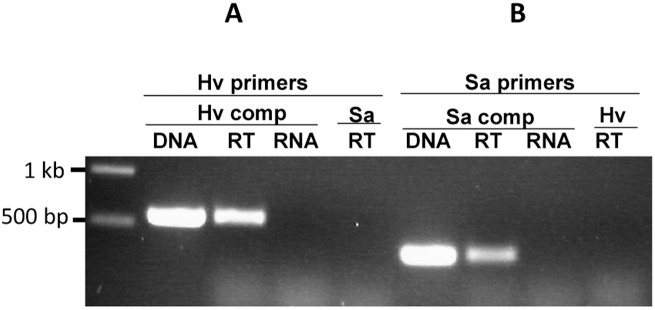
Detection of transcription of *Hfx*. *volcanii* and *S*. *acidocaldarius aglB* in complemented *Mc*. *maripaludis* Δ*aglB-14-9* mutant cells. Total RNA was purified from complemented cells grown for three successive transfers in nitrogen-free medium supplemented with alanine to allow transcription of the *aglB* gene from the *nif* promoter in the complementation vector. In both cases, the *aglB* was synthesized using *Mc*. *maripaludis* codon preferences. **A.** *Mc*. *maripaludis* Δ*aglB-14-9* mutant complemented with *Hfx*. *volcanii aglB*. Purified RNA with (RT) or without (RNA) a reverse transcription step was used as template using RT primers for *Hfx*. *volcanii aglB* (Table 2). Standard PCR with the same primers was used with pKJ1251 as template for amplicon size verification (DNA). The *Hfx*. *volcanii aglB* RT-PCR primers were also used in reactions with RNA isolated from *Mc*. *maripaludis* Δ*aglB-14-9* mutant complemented with *S*. *acidocaldarius aglB* (Sa RT). **B.** *Mc*. *maripaludis* Δ*aglB-14-9* mutant complemented with *S*. *acidocaldarius aglB*. Purified RNA with (RT) or without (RNA) a reverse transcription step was used as template using RT primers for *S*. *acidocaldarius aglB* (Table 2). Standard PCR with the same primers was used with pKJ1240 as template for amplicon size verification (DNA). The *S*. *acidocaldarius aglB* RT-PCR primers were also used in reactions with RNA isolated from *Mc*. *maripaludis* Δ*aglB-14-9* mutant complemented with *Hfx*. *volcanii aglB* (Hv RT).

The other two failed complementations used the *aglB*s from *S*. *acidocaldarius* and *Hfx*. *volcanii* (Fig 4D and 4E). These two organisms are much more distantly related to *Mc*. *maripaludis* than *Mc*. *voltae* and *Mtc*. *thermolithotrophicus*, with *S*. *acidocaldarius* being a member of a different phylum, the Crenarchaeota. These organisms were chosen as, along with *Mc*. *maripaludis*, they are two of the best studied in terms of various aspects of the *N*-glycosylation pathways with information available on glycan structure, linking sugars, dolichol carriers and AglB [1]. For these complementation studies, the *Hfx*. *volcanii* and *S*. *acidocaldarius aglB* were synthesized using *Mc*. *maripaludis* codon preferences and with the addition of a C-terminal FLAG tag. Using the same conditions that resulted in successful complementation as reported above, no indication of successful complementation of the Δ*aglB-14-9* strain was observed by western blot using either the *S*. *acidocaldarius* or *Hfx*. *volcanii aglB*. While attempts to detect synthesis of the AglBs from *Hfx*. *volcanii* and *S*. *acidocaldarius* with anti-FLAG antiserum in the complemented Δ*aglB-14-9* strain lysates were unsuccessful (not shown), RT-PCR demonstrated that the mRNA for each *aglB* was present in the respective complementations (Fig 6). Expression of archaeal AglBs in foreign hosts can be problematic. While good expression of *Mc*. *voltae* AglB in *E*.*coli* was reported [2], Taguchi et al. (2016) were unable to produce more than a trace of AglB from *Sulfolobus solfataricus* or *Pyrobaculum calidifontis* in *E*.*coli* [24]. Expression of active AglB from three different extreme halophiles in *Hfx*. *volcanii* was achieved although to greatly varying degrees [34]. The *aglB* from *Hfx*. *volcanii* was also cloned directly from genomic DNA without *Mc*. *maripaludis* codon optimization but it too could not rescue oligosaccharyltransferase activity in the Δ*aglB-14-9* strain (not shown).

In *Hfx*. *volcanii*, the *N*-linked glycosylation system is very complicated. Completely different glycans are found depending on the salt content of the growth medium. At high salt (3.4M NaCl), a pentasaccharide (mannose-1,2-[methyl-*O*-4-]GlcA-β-1,4-galacturonic acid-α1,4-GlcA-β1,4-glucose-β1-Asn) is made [9]. The first four sugars are assembled on a single dolichol carrier and transferred by AglB while the terminal mannose loaded onto a separate dolichol carrier is transferred to the Asn-bound tetrasaccharide by AglS [23,25]. Here, the linking sugar is unusual in being glucose, rather than an acetylated sugar. At growth in low salt (1.75M NaCl), a tetrasaccharide consisting of a sulfated hexose, two hexoses and a rhamnose is found [64]. Intriguingly, only a single *aglB* gene is reported for *Hfx*. *volcanii* but this AglB is only required for the transfer of the pentasaccharide but not the low salt tetrasaccharide [36]. The oligosaccharyltransferase responsible for transfer of the low salt glycan is not yet identified. The percent identity and similarity of the *Hfx*. *volcanii* AglB to that of *Mc*. *maripaludis* is much lower (17.9/ 31.0%) than that shown by the *Mc*. *voltae* and *Mtc*. *thermolithotrophicus* enzymes and the halophile enzyme is much longer. In addition, the DK/MI motif is completely different in halophiles and methanogens. The DK motif has been shown to be catalytically important both in yeast Stt3 [65] as well as the AglBs of *P*. *furiosus* and *A*. *fulgidus* [33,56]. In *Hfx*. *volcanii*, the sequence is the modified DM version of the motif, **D**WQ**M**ASTDA**K**. Methanococci have the MI motif at the corresponding position in AglB believed to perform the same role as the DK/DM motif and in *Mc*. *maripaludis* the MI motif is **M**TS**I**ASV**W**. In PglB of *Campylobacter jejuni*, the MI motif (**M**SL**I**FST**V**) has been shown by mutational analysis to be important for catalytic activity with only chemically similar amino acids tolerated at the conserved positions [66]. Given that the chemical properties of the DK, DM and MI side chains are considerably different and the effect this has on enzyme function is not understood [57], it is possible the replacement of the DK/DM motif with the MI motif affects recognition of substrates by the different AglBs. Furthermore, the catalytic site of all AglBs is in the C-terminal domain predicted to be located external to the cell. Consequently, this region may be specifically adapted to the high external salt environment of *Hfx*. *volcanii* and not properly folded in the 2% NaCl-containing medium used for *Mc*. *maripaludis*. It is also possible that the halophile AglB was misfolded and either degraded or did not insert properly into the cytoplasmic membrane, although the membrane lipids of *Hfx*. *volcanii* are diether types like that in *Mc*. *maripaludis* [67]. Other less likely, in our opinion, possibilities for the failure of the *Hfx*. *volcanii* AglB complementation can also be envisioned. One is the difference in the linking sugar in the glycans of *Hfx*. *volcanii* and *Mc*. *maripaludis*. However, it is known that the *Hfx*. *volcanii aglB* can be replaced with *aglB* from different extreme halophiles that can transfer glycans with either a hexose or *N*-acetylhexuronic acid as a linking sugar so one would expect that the AglB from *Hfx*. *volcanii* would accommodate a GalNAc linking sugar. Another unlikely reason for the failure of the *Hfx*. *volcanii* AglB to complement could lie in the nature of the dolichol carrier, but the *Hfx*. *volcanii* carrier is C_55_-C_60_ dolichol phosphate with saturated isoprenes at the α and ω positions [27] and in *Mc*. *maripaludis* it is a C_55_ dolichol phosphate that has two sites of saturation, presumed to be the α and ω positions too [2].

The other failed complementation occurred with the *S*. *acidocaldarius* AglB. In *S*. *acidocaldarius*, the *N*-linked glycan is a complex tri-branched hexasaccharide containing the unusual sulfated sugar, sulfoquinovose and with a linking sugar of *N*-acetylglucosamine [14]. Many possible reasons for the failure can be presented. Obviously, the active site of this AglB would be facing an extremely acidic environment and the enzyme would need to be adapted for the high growth temperature as well, both not found in the growth conditions of *Mc*. *maripaludis*. Even with an AglB from the much more closely related hyperthermophile *Mcc*. *jannaschii*, we were unable to show complementation. Furthermore, in its native environment AglB of *S*. *acidocaldarius* would be inserted into a cytoplasmic membrane comprised of tetraether lipids, including ones that could contain up to 4 cyclopentane rings [67,68]. Cytoplasmic membranes composed solely of tetraether lipids are thought to form a lipid monolayer and not the lipid bilayer seen in bacteria or in archaea which have diether lipids [69,70]. As with the *Hfx*. *volcanii* AglB, the one from *S*. *acidocaldarius* shows low sequence identity and similarity to that of *Mc*. *maripaludis* (19.9%/34.1%). The *S*. *acidocaldarius* AglB has the DK motif (**D**IA**K**) rather than the MI motif of *Mc*. *maripaludis* AglB. Furthermore, there are potentially significant differences in the substrates encountered. The linking sugar in the glycan of *S*. *acidocaldarius* is GlcNAc and not GalNAc as in *Mc*. *maripaludis*. In addition, the dolichol carrier normally recognized by AglB in *S*. *acidocaldarius* was recently shown to be an unusual, short (C_45_) dolichol pyrophosphate [30] and not the dolichol phosphate initially believed to serve as the glycan lipid carrier [28] and this could cause recognition problems for the *S*. *acidocaldarius* AglB towards the *Mc*. *maripaludis* dolichol carrier, identified as C_55_ dolichol phosphate [2].

## Conclusions

This study extends to methanogens the examination of AglB promiscuity previously reported in *in vivo* experiments with extreme halophiles [34] and *in vitro* with the hyperthermophiles *A*. *fulgidus* and *P*. *furiosus* [33]. We have shown the ability of *Mc*. *voltae* AglB to transfer glycans of similar but different composition, including glycans with either GlcNAC or GalNAc as the linking sugar. We have also identified for the first time the AglB from the thermophilic methanogen *Mtc*. *thermolithotrophicus* and demonstrate that it can functionally replace the oligosaccharyltransferase activity in the *Mc*. *maripaludis* Δ*aglB* mutant. As there are currently no published data on the nature of *N*-linked glycans in *Mtc*. *thermolithotrophicus*, it is not possible to state how different the *Mc*. *maripaludis* glycan is from that naturally transferred by the *Mtc*. *thermolithotrophicus* AglB. We have begun studies to determine the *N*-glycan attached to archaellins in this thermophilic methanogen to address this issue. Attempts to complement using AglBs from more distantly related and more extremophilic archaea for which data on *N*-linked glycosylation systems is well known were unsuccessful, although the possible reasons for this are many. The accumulation of data on the relaxed nature of substrates accepted by various AglBs will aid in any efforts to develop archaea as platforms for glycoengineering [44].

## Supporting Information

S1 Fig Alignment of AglBs from *Mc*. *maripaludis* and *Mc*. *voltae* using EMBOSS Needle. (DOCX)

S2 Fig Alignment of AglBs from *Mc*. *maripaludis* and *Mtc*. *thermolithotrophicus* using EMBOSS Needle. (DOCX)

S3 Fig Alignment of AglBs from *Mc*. *maripaludis* and *Mcc*. *jannaschii* using EMBOSS Needle. (DOCX)

S4 Fig Alignment of AglBs from *Mc*. *maripaludis* and *Hfx*. *volcanii* using EMBOSS Needle. (DOCX)

S5 Fig Alignment of AglBs from *Mc*. *maripaludis* and *S*. *acidocaldarius* using EMBOSS Needle. (DOCX)
