# Supplementary material for: Complementation of an aglB Mutant of Methanococcus maripaludis with Heterologous Oligosaccharyltransferases
Source: PLoS One. 2016 Dec 1;11(12):e0167611. doi: 10.1371/journal.pone.0167611 (PMC5131992; doi:10.1371/journal.pone.0167611)
Supplement: S1 Fig — (DOCX) [file pone.0167611.s001.docx]

S1 Fig Alignment of AglB from *Mc. maripaludis* and *Mc. voltae*, using EMBOSS Needle.

maripaludis 1 --------------------MGEFLNKVSDFFKKNEKIKIILILLFIGMM 30

..:|.....| ||.:..|.|||::|:..:

voltae 1 MTENNEKVKNSDSANNQSSKNSKFNFNFED--KKVKCAKTILIIIFLAFL 48

maripaludis 31 SFQIRAQTADMAFTDNSYLQDMFSDDNGRMYLTALDPYYYLRMTENYVNN 80

|||:|||||||.||.|....|:|||||||||||||||||||||:|||:.|

voltae 49 SFQMRAQTADMGFTTNEQYLDVFSDDNGRMYLTALDPYYYLRMSENYLEN 98

maripaludis 81 DYSNVGETTVGIDGENIPYDTIQYAPPGREAGLVSALSIATVLVYSVWNS 130

.:: |:|...|||:.:|:|:.:|.|.|..| ..:.||:.||.||.||::

voltae 99 GHT--GDTLKNIDGQQVPWDSYKYGPTGARA-TFNLLSVVTVWVYQVWHA 145

maripaludis 131 IDSTVTIMNAAFWVPAIMSIFLGIPVFFIVRRNTASNIGGLVGALLLISS 180

:|||||:||||||||||:|:||..|:||.|||.|:|:|||.|.|:|...|

voltae 146 MDSTVTLMNAAFWVPAILSMFLITPIFFTVRRITSSDIGGAVAAILASLS 195

maripaludis 181 PSLLYKTSAGFSDTPIFEILPLLFIVWMIMEAIH-EQENSKKSGIFGGIA 229

||:..||.||||||||.||||||||||.|:|||| .:|.:.||.|:|.:|

voltae 196 PSIFVKTVAGFSDTPILEILPLLFIVWFIIEAIHYSKEKNYKSLIYGLLA 245

maripaludis 230 AILIGLYPMMWSGWWYAFDITAGFLVLYTAYEYL--------TKSKN--- 268

.:::.|||.|||.|||.:.|...|||:|..|:.: |||||

voltae 246 TLMLALYPFMWSAWWYGYYIVIAFLVIYAIYKGISYNSIAKYTKSKNNNH 295

maripaludis 269 ----------LKNVITTSLITLVGGAILVSLSTGLSGFINWILSPIGFTV 308

:.|::..|.:.::|||:|::...|:|..:|.:.:|:.:..

voltae 296 KDKIESEKLEMLNILKISGLFIIGGAVLITALYGVSTTMNALQAPLNYLG 345

maripaludis 309 INEATKITGWPNVYMTVSELAIPTVTDIIENSVGNIWLLIAGISGILLSF 358

::|.:..||||||..|||||...::.:||.:|:|:|.|...|:.||.||.

voltae 346 LDEVSSQTGWPNVLTTVSELDTASLDEIISSSLGSIHLFAIGLIGIFLSL 395

maripaludis 359 VSFKHD-----------KQKIDIKYALYLTLWLIATVYAATKGIRFVALM 397

|:.. .:|:||||||.|.:|...|..||:||:||||||

voltae 396 --FRKVLTPVKQISNGLAEKLDIKYALLLIIWFAVTFLAASKGVRFVALM 443

maripaludis 398 TPALAIGIGIFAGQIENIIK-RYEKKVEYILYPVIGILSVITLIKY---- 442

.|.|:||:|||.|.||..|| ..:||.||:.||.|.|:.:..|...

voltae 444 VPPLSIGVGIFVGFIEQFIKNNLDKKYEYVAYPTIAIIVLYALFTIYRAD 493

maripaludis 443 GGELFNILVPTTYVPIAVYLSIIALLVLAVYKIIDIISE--KEQAVKKVF 490

..:|..:|:|:.|||||..:.:.:|.||.:||:.::|:| |:..:.|:|

voltae 494 SADLVRMLLPSNYVPIAEGIMLASLAVLIIYKVAELIAESNKKLVMNKIF 543

maripaludis 491 GILLAFMLVFPSMAAAVPFYTAPTMNNGWMDSLSWIKSETPENSVVTCWW 540

.||||..|:.|::|..||||:.||.|:||.:||.||.::||.||||||||

voltae 544 MILLAIGLITPTIATIVPFYSVPTYNDGWGESLEWINTQTPNNSVVTCWW 593

maripaludis 541 DNGHIYTWATRKMVTFDGGSQNTPRAYWVGHAFSTSDENLSVGILRMLAT 590

||||||||.|.:||||||.|||||||||||.|||||:|:|:.||.||||:

voltae 594 DNGHIYTWKTDRMVTFDGSSQNTPRAYWVGRAFSTSNESLANGIFRMLAS 643

maripaludis 591 SGDSAYDDDSILIKKTGSIKDTVDILNKILPLTRTEAKASLVN-NYDLTD 639

|||.||..||:|||||||||:|||:||:|||||:::|:.:|.| :|..||

voltae 644 SGDKAYTTDSVLIKKTGSIKNTVDVLNEILPLTKSDAQKALKNSSYKFTD 693

maripaludis 640 AEAEEVLDLTHPKVTNPDYLITYNRMTSIASVWSMFGNWNFSLPASTENS 689

.|..|:||.|||||||||||||||||||||||||.||||:|:|||.|..|

voltae 694 TEVSEILDATHPKVTNPDYLITYNRMTSIASVWSYFGNWDFNLPAGTSRS 743

maripaludis 690 DREMGYYQQLGGSAQDINGTTVVYIPLQETDSYRVINILEITDSEIKSAN 739

:||.|.:|.|...|.:||.|.:|...:|:|..|.:..::|:.:..:..|.

voltae 744 EREAGSFQGLQTYATNINDTLIVRSLIQQTAEYNIYTLIEVRNETLTGAM 793

maripaludis 740 AVIDSNNQTSMQSPNFHKLILKVNGN----VYEQETNENGDYSEIVRLEK 785

..:.::.|...|..|.||:.|.||.| :|....:.:|..|.:::::|

voltae 794 MAVTNDGQMQTQQLNMHKVKLMVNENGKSKMYNSLADPDGQLSLLIKVDK 843

maripaludis 786 LS----DGT----YQVYAWVSSKNLEDSIYTKLHFLDGYGLEKISLEKES 827

.| ||: |...:|:::.|||||:|:||||.||.||:.|.|||||

voltae 844 NSIIGTDGSNNPVYSSSSWMATANLEDSVYSKLHFFDGEGLDTIKLEKES 893

maripaludis 828 VDPTSYGIQPGFKVYSVDYGTDYLN 852

:|||:.|:||||||:||||| :|..

voltae 894 LDPTANGVQPGFKVFSVDYG-NYSK 917
