# Supplementary material for: Complementation of an aglB Mutant of Methanococcus maripaludis with Heterologous Oligosaccharyltransferases
Source: PLoS One. 2016 Dec 1;11(12):e0167611. doi: 10.1371/journal.pone.0167611 (PMC5131992; doi:10.1371/journal.pone.0167611)
Supplement: S2 Fig — (DOCX) [file pone.0167611.s002.docx]

S2 Fig. Alignment of AglB from *Mc. maripaludis* and *Mtc. thermolithotrophicus*, using EMBOSS Needle.

maripaludis 1 MGEFLNKVSDFFKKNEKIKIILILLFIGMMSFQIRAQTADMAFTDNSYLQ 50

|||||||.|:|||:.|.||:.||:|.:|::|||:|||.|||.||::..|:

thermolithotr 1 MGEFLNKFSEFFKRRENIKVFLIILVLGLVSFQLRAQPADMGFTNDVQLK 50

maripaludis 51 DMFSDDNGRMYLTALDPYYYLRMTENYVNNDYSNVGETTVGIDGENIPYD 100

:||:|:||||||.|||||:|||:|||| :|:.::|||...:||:.:|||

thermolithotr 51 NMFADENGRMYLIALDPYWYLRLTENY--HDHGHLGETLKEVDGKLVPYD 98

maripaludis 101 TIQYAPPGREA-GLVSALSIATVLVYSVWNSIDSTVTIMNAAFWVPAIMS 149

||||||||..| ..|..||:.|:.:||:|:|.|||||:|||||||||:|.

thermolithotr 99 TIQYAPPGHPAPDKVPILSLVTLGIYSIWHSFDSTVTLMNAAFWVPALMG 148

maripaludis 150 IFLGIPVFFIVRRNTASNIGGLVGALLLISSPSLLYKTSAGFSDTPIFEI 199

:.||||:||::||:|:||:||:||||::.|||:|||||||||:||||||:

thermolithotr 149 MLLGIPIFFMIRRSTSSNVGGVVGALIIASSPALLYKTSAGFADTPIFEV 198

maripaludis 200 LPLLFIVWMIMEAIHEQENSKKSGIFGGIAAILIGLYPMMWSGWWYAFDI 249

||:|||.|.||||||.|.|.|||.||..:|.:||.|||.||:|||||::|

thermolithotr 199 LPILFIAWFIMEAIHYQNNLKKSIIFTALATLLIALYPRMWAGWWYAYNI 248

maripaludis 250 TAGFLVLYTAYEYLTK----SKNLKNVITTSLITLVGGAILVSLSTGLSG 295

..|||::|.||..:.| |.|.||:::.:.:.::||.:|||:..|::.

thermolithotr 249 VTGFLIIYLAYLCVVKKSHESDNFKNLLSITGLFVLGGGLLVSVFYGINS 298

maripaludis 296 FINWILSPIGFTVINEATKITGWPNVYMTVSELAIPTVTDIIENSVGNIW 345

|||.:|||:|||.|...:..|||||||.|||||:..:...|:.||||:||

thermolithotr 299 FINGVLSPVGFTTIKVVSHATGWPNVYTTVSELSATSFNSIVNNSVGSIW 348

maripaludis 346 LLIAGISGILLSFVSFKHDKQKIDIKYALYLTLWLIATVYAATKGIRFVA 395

|.|.||.|::.||:|.:|.|::.|:||.|.|.|||.||.||||||:||:.

thermolithotr 349 LFIVGILGVISSFISLRHGKKEFDVKYGLLLILWLAATGYAATKGVRFIG 398

maripaludis 396 LMTPALAIGIGIFAGQIENIIKRYEKK-VEYILYPVIGILSVITLIKYGG 444

||||.|||||||.|||:|||:|..:.. ::|.||||:||:|::.:....|

thermolithotr 399 LMTPPLAIGIGILAGQLENIVKISKSNLIKYSLYPVVGIVSLLFIATNAG 448

maripaludis 445 ELFNILVPTTYVPIAVYLSIIALLVLAVYKIIDII-SEKEQAVKKVFGIL 493

::..|::|:||||||.|..|..::||.:|||.||| |::|...||:|.:|

thermolithotr 449 KIPEIILPSTYVPIAAYGLISLVVVLLLYKIADIISSDRENKFKKLFSVL 498

maripaludis 494 LAFMLVFPSMAAAVPFYTAPTMNNGWMDSLSWIKSETPENSVVTCWWDNG 543

|...||.||:|:|||.|||||.|:||.:||.|||:|||.|||:|||||||

thermolithotr 499 LGIALVLPSLASAVPLYTAPTFNDGWKESLDWIKTETPNNSVITCWWDNG 548

maripaludis 544 HIYTWATRKMVTFDGGSQNTPRAYWVGHAFSTSDENLSVGILRMLATSGD 593

|||||||||||||||||||:|||||||.|:|||:||||||||||||||||

thermolithotr 549 HIYTWATRKMVTFDGGSQNSPRAYWVGKAYSTSNENLSVGILRMLATSGD 598

maripaludis 594 SAYDDDSILIKKT-GSIKDTVDILNKILPLTRTEAKASLVNNYDLTDAEA 642

.|::.|.||:.|| .|:.:||.|||:|||:::::|...|||.|:||:.||

thermolithotr 599 KAFEKDGILMNKTNNSVGETVKILNEILPVSKSKAYNILVNKYNLTEKEA 648

maripaludis 643 EEVLDLTHPKVTNPDYLITYNRMTSIASVWSMFGNWNFSLPASTENSDRE 692

.|||:.|||:..||||||||||||.||.||||||||:||:|.:|.|.:||

thermolithotr 649 LEVLNATHPENPNPDYLITYNRMTDIAPVWSMFGNWDFSMPPNTPNEERE 698

maripaludis 693 MGYYQQLGGSAQDINGTTVVYIPLQETDSYRVINILEITDSEIKSANAVI 742

.|||.:|.|.....|||..:.:|:|:|.:|.|:|::.|.:|.|.|.:...

thermolithotr 699 SGYYTKLRGEGVLNNGTLFIRVPIQKTQNYAVMNVILIQNSSIASYDLTY 748

maripaludis 743 DSNNQTSM--QSPNFHKLILKVNGNVYEQETNENGDYSEIVRLEKLSDGT 790

::|..|.| ::..|||:|:|...||||:|.|:||..||||||..:.:..

thermolithotr 749 NTNTGTVMSEKTTGFHKVIVKNGNNVYEKEFNKNGTISEIVRLVPVGNNQ 798

maripaludis 791 YQVYAWVSSKNLEDSIYTKLHFLDGYGLEKISLEKESVDPTSYGIQPGFK 840

|..|.|::|:||||||||:|||||||||:.|.|.|||.|||:||||||||

thermolithotr 799 YYTYVWIASRNLEDSIYTRLHFLDGYGLKHIKLVKESQDPTNYGIQPGFK 848

maripaludis 841 VYSVDYGTDYLN 852

||.||||.||||

thermolithotr 849 VYEVDYGIDYLN 860
