# Supplementary material for: Complementation of an aglB Mutant of Methanococcus maripaludis with Heterologous Oligosaccharyltransferases
Source: PLoS One. 2016 Dec 1;11(12):e0167611. doi: 10.1371/journal.pone.0167611 (PMC5131992; doi:10.1371/journal.pone.0167611)
Supplement: S3 Fig — (DOCX) [file pone.0167611.s003.docx]

S3 Fig. Alignment of AglB from *Mc. maripaludis* and *Mcc. jannaschii*, using EMBOSS Needle.

maripaludis 1 -------MGEFLNKVSDFFKKNEKIKIILILLFIGMMSFQIRAQTADMAF 43

|...|.|:::|||:...||:.||:|.:..:|||:|||||||.|

jannaschii 1 MYIKVKLMSNALEKINNFFKEKSWIKVFLIILMLMFVSFQLRAQTADMKF 50

maripaludis 44 T-DNSYLQDMFSDDNGRMYLTALDPYYYLRMTENYVNNDYSNVGETTVGI 92

. ||.:|:|||||::|||||.|||||||||::||..|| .:.|:|...:

jannaschii 51 AQDNEFLKDMFSDEHGRMYLLALDPYYYLRLSENLYNN--GHCGDTIKVV 98

maripaludis 93 DGENIPYDTIQYAPPGR----EAGLVSALSIATVLVYSVWNSIDSTVTIM 138

||:..|||..||||||. |..:: .:||:.:|.:|:|||.|||||

jannaschii 99 DGKETPYDLYQYAPPGHPLPWEPPVI---CLATLAIYYIWHSIDLTVTIM 145

maripaludis 139 NAAFWVPAIMSIFLGIPVFFIVRRNTASNIGGLVGALLLISSPSLLYKTS 188

||||||||::.:.||||::|:|||.|.|||||:.||:.|||:|.|||||.

jannaschii 146 NAAFWVPAVLGMLLGIPIYFVVRRVTNSNIGGIAGAIALISAPGLLYKTC 195

maripaludis 189 AGFSDTPIFEILPLLFIVWMIMEAIHEQENS---KK-------------- 221

|||:||||||:||:|||||.|:|:||.||.: ||

jannaschii 196 AGFADTPIFEVLPILFIVWFILESIHSQEKTALFKKDLKNPISLFVIAAL 245

maripaludis 222 -------------SG----------------------------------- 223

||

jannaschii 246 IIELIIGAYLNIASGESVVIASILFYTVSLAFILAGLIIAGIKKLKGNEL 295

maripaludis 224 ---IFGGIAAILIGLYPMMWSGWWYAFDITAGFLVLYTAYEYLTKSK--- 267

:|..:|.||..:.|.||..|||.||:...|||:|.....|.||:

jannaschii 296 EFELFALLAVILTAVSPKMWGAWWYGFDVITAFLVIYIIALALLKSQVKI 345

maripaludis 268 -------NLKNVITTSLITLVGGAILVSLSTGLSGFINWILSPIGFT-VI 309

||||::..|:..:.|..:|:....|:...|:.|.||:|:. ::

jannaschii 346 KEFINIGNLKNIVYLSIFYIFGSFVLLVAIYGMGIAISPITSPLGYNQIL 395

maripaludis 310 NEATKITGWPNVYMTVSELAIPTV-TDIIENSVGNIWLLIAGISGILLSF 358

:..|:.|||||||.||:|||.|:. ::|..|::|:..:.|.||.||||||

jannaschii 396 STYTQTTGWPNVYTTVAELAKPSSWSEIFTNAIGSDTIAIVGILGILLSF 445

maripaludis 359 VSFKHDKQKIDIKYALYLTLWLIATVYAATKGIRFVALMTPALAIGIGIF 408

:|.:::|.|:||||::.|.:||..|:|||||||||.||.||.||||:|||

jannaschii 446 LSLRYEKVKLDIKYSILLAIWLAVTLYAATKGIRFAALATPPLAIGLGIF 495

maripaludis 409 AGQIENIIKRYEKKVEYILYPV---IGILSVITLIKYGGELFNILVPTTY 455

.||:|..:| .|.:..::.: .||..::.|.||..::..||:||||

jannaschii 496 VGQLERFLK---MKSDIAIFGIGIPAGIFGLLILSKYSAKISQILLPTTY 542

maripaludis 456 VPIAVYLSIIALLVLAVYKIIDIIS---EKEQAVKKVFGILLAFMLVFPS 502

|||..|..:|.|.:||:|||.|||| :|::.:.||..:||...:|.|.

jannaschii 543 VPIIAYGFLIVLALLAIYKISDIISTLNDKKETIIKVSTLLLCIGVVIPP 592

maripaludis 503 MAAAVPFYTAPTMNNGWMDSLSWIKSETPENSVVTCWWDNGHIYTWATRK 552

::|.|||..|||.||||.:.|.|||:.||.|||:|||||||||||:..|:

jannaschii 593 LSAVVPFSVAPTFNNGWKEGLDWIKANTPNNSVITCWWDNGHIYTYEARR 642

maripaludis 553 MVTFDGGSQNTPRAYWVGHAFSTSDENLSVGILRMLATSGDSAYDDDSIL 602

||||||||||:|||||||.||:||:||||:||:||||||||.|:...|:|

jannaschii 643 MVTFDGGSQNSPRAYWVGRAFATSNENLSIGIIRMLATSGDEAFKKGSVL 692

maripaludis 603 IKKT-GSIKDTVDILNKILPLTRTEAKASLVNNYDLTDAEAEEVLDLTHP 651

:..| .::..||.|||:|||:.|::|...|...|.|:|.:|:.||:.|||

jannaschii 693 MNFTHNNVSKTVKILNEILPVDRSKAYDILTKKYGLSDKKAKLVLNATHP 742

maripaludis 652 KVTNPDYLITYNRMTSIASVWSMFGNWNFSLPASTENSDREMG------- 694

:..||||||||||||.||.||||||.||||||.:|.|..||.|

jannaschii 743 EHPNPDYLITYNRMTDIAPVWSMFGFWNFSLPPNTPNDKREKGAFFKGTA 792

maripaludis 695 YYQQLGGSAQDINGTTVVYIPLQETDSYRVINILEITDSEIKSANAVIDS 744

||...|....::|..|..|:.| ||...|:.:.::..|

jannaschii 793 YYLGNGTILANVNVYTYSYVTL--------INSTNISTAIVQKIN----- 829

maripaludis 745 NNQTSMQSPNFHKLILKVNGNVYEQETNENGDYSEIVRLEKLSDGTYQVY 794

.....:.:...|||.:|....|.|...|::|..||.:|:| :|| :.|

jannaschii 830 GQAKIIGTFKIHKLYIKTPLGVKELVLNKDGQLSEFIRIE--ADG--RGY 875

maripaludis 795 AWVSSKNLEDSIYTKLHFLDGYGLEKISLEKESVDPTSYGIQPGFKVYSV 844

||::::|||||||.||||||||||:.|.|.|.::|||.:|||||||:|.|

jannaschii 876 AWLATRNLEDSIYAKLHFLDGYGLKHIKLVKATIDPTDFGIQPGFKIYKV 925

maripaludis 845 DYGTDYLN 852

|||||||.

jannaschii 926 DYGTDYLK 933
