# Supplementary material for: Complementation of an aglB Mutant of Methanococcus maripaludis with Heterologous Oligosaccharyltransferases
Source: PLoS One. 2016 Dec 1;11(12):e0167611. doi: 10.1371/journal.pone.0167611 (PMC5131992; doi:10.1371/journal.pone.0167611)
Supplement: S4 Fig — (DOCX) [file pone.0167611.s004.docx]

S4 Fig. Alignment of AglB from *Mc. maripaludis* and *Hfx. volcanii*, using EMBOSS Needle.

maripaludis 1 MGEFLNKVS--------DFFKKNEKIKIILILLFIGMMSFQIRAQTADMA 42

|.:...|.| |::.......|||::|:|.:.|:

volcanii 1 MSDEQTKYSPSIAELARDWYHIPVLSTIILVMLWIRLRSY---------- 40

maripaludis 43 FTDNSYLQDMFSDDNGRMYLTALDPYYYLRMTENYVNNDYSNVGETTVGI 92

:::::: |.::.:..|.:|:||..|..|.|..:

volcanii 41 ---DAFIRE------GTVFFSGNDAWYHLRQVEYTVRNWPA--------- 72

maripaludis 93 DGENIPYDTIQYAPPGREAG----LVSALSIATVLVYSVWNSIDSTVTIM 138

.:|:|.....|.||.|| :...|.....||..:.: .|:..:.

volcanii 73 ---TMPFDPWTEFPFGRTAGQFGTIYDQLVATAALVVGLGS--PSSDLVA 117

maripaludis 139 NAAFWVPAIMSIFLGIPVFFIVRRNTASNIGGLVGALLLISSP-SLLYKT 187

.:....||:......||.:.|.:| ....:|||.||::|:..| :.|.:.

volcanii 118 KSLLVAPAVFGALTVIPTYLIGKR-LGGRLGGLFGAVILMLLPGTFLQRG 166

maripaludis 188 SAGFSDTPIFEILPLLFIVWMIMEAIHEQENSK----------------- 220

..||:|..|.|...:.|.|..||.|:...:..|

volcanii 167 LVGFADHNIVEPFFMGFAVLAIMIALTVADREKPVWELVAARDLDALREP 216

maripaludis 221 -KSGIFGGIAAILIGLYPMMWSGWWYAFDITAG----FLVLYTAYEYLTK 265

|..:..|:|..: .||| |....:..| ||||..|.:|: :

volcanii 217 LKWSVLAGVATAI-----YMWS--WPPGILLVGIFGLFLVLKMASDYV-R 258

maripaludis 266 SKNLKNVITTSLITLVGGAILVSLSTGLSGFINWILSPI---GFTVI--- 309

.::.:: |...|||.::: |||..|| || ||.|.

volcanii 259 GRSPEH-------TAFVGAISMTV-TGLLMFI-----PIEEPGFGVTDFG 295

maripaludis 310 --------------------------NEATK------ITGWPNVYMTVSE 327

|:..: :.|...|.:.:..

volcanii 296 FLQPLFSLGVALGAVFLAALARWWESNDVDERYYPAVVGGTMLVGIVLFS 345

maripaludis 328 LAIPTVTDIIENSVGNIWLLIAGIS-GILLSFVS----------FKHDKQ 366

|.:|:|.| |:...:|...|.| |.....:| .:.:.|

volcanii 346 LVLPSVFD----SIARNFLRTVGFSAGAATRTISEAQPFLAANVLQSNGQ 391

maripaludis 367 ----KIDIKYAL-YLT-----LWLIATV-----------YA----ATKGI 391

:|..:|.. :.| :||:|.. || |..|:

volcanii 392 TAVGRIMSEYGFTFFTGALAAVWLVAKPLVKGGNSRKIGYAVGSLALIGV 441

maripaludis 392 RFVALMTPALAIGIGIFAGQIENIIKRYEKKVEYILYPVIGILSVITLI- 440

.| :.|||..|||...| || |.:..|:::|.:

volcanii 442 LF---LIPALPAGIGSALG------------VE----PSLVSLTIVTALI 472

maripaludis 441 -------KYGGELFNILV--------PTTYVPIAVYLSIIALLVLA---- 471

.|..|...:|| ..|.|....||:::..::.|

volcanii 473 VGAVMQADYESERLFVLVWAAIITSAAFTQVRFNYYLAVVVAVMNAYLLR 522

maripaludis 472 ------------VYKIIDIISEKEQAVKKVFGILLAFMLVFP-------- 501

|.:..||...:..||.....::|..:|:.|

volcanii 523 EALGIDFVGLANVERFDDISYGQVAAVVIAVLLILTPVLIIPIQLGNGGV 572

maripaludis 502 SMAAAVPFYTAPTMNNGWMDSLSWIKSETPENSV---------------- 535

|..|.....|.|.....|..||:|:::.||....

volcanii 573 SQTAMQASQTGPGTVTQWDGSLTWMQNNTPAEGEFGGESNRMEYYGTYEY 622

maripaludis 536 ------------VTCWWDNGHIYTWATRKMVT---FDGGSQNTPRAYWVG 570

|..|||.||..|....::.. |.||: |..|.::

volcanii 623 TDDFDYPDGAYGVMSWWDYGHWITVLGERIPNANPFQGGA--TEAANYL- 669

maripaludis 571 HAFSTSDENLSVGILRMLATSGDSAYDDDSILIKKTGSIKDTVDILNKIL 620

...||..:..: |.:.||....|.:..: .|| .

volcanii 670 ---LAEDEQQAESV---LTSMGDDGEGDQTRYV--------MVD-----W 700

maripaludis 621 PLTRTEAKASLVN-NYDLTDAEAEEVLDLTHPKVTNPDYLITYNRM---- 665

.:..|:||.|... .|| ...::..|: ||.|

volcanii 701 QMASTDAKFSAPTVFYD-------------ESNISRSDF---YNPMFRLQ 734

maripaludis 666 -----TSIASVWSMFGNWNFSLPASTENSDREMGYYQQL-------GGSA 703

|::|:..|: ::..||:.| .|||

volcanii 735 EQGEQTTVAAASSL----------------KDQRYYESLMVRLYAYHGSA 768

maripaludis 704 QD-------------INGTTVVYIPLQETDSYRVINILEITDSEIKSAN- 739

:: .:|:|...:...:..:.|..:.:...:..: ||

volcanii 769 REASPIVVDWEERTSADGSTTFRVTPSDGQAVRTFDNMSAAEEYV--AND 816

maripaludis 740 ----------------------AVIDSNNQTSMQSPNFHKLILKVNGNVY 767

.::.|:|.::::|.::.:.::. .||.|

volcanii 817 PTSQIGGIGTFPEERVSALEHYRLVKSSNSSALRSGSYQRSLIS-EGNTY 865

maripaludis 768 --EQETNENGDYSEIVRLEKLSDGTYQ---------VYAWVSSKNL---E 803

:.:.....:.:.:...|::...|.. |.|.|..::| .

volcanii 866 GLQPQALVPNNPAWVKTFERVPGATVDGSGAPANTTVTARVQMRDLTTGT 915

maripaludis 804 DSIYTKLHFLDGYGLEKISLEKESVDPTSYGIQPGFKVYSVDYGTDYLN- 852

:..||:....|..|...::|...:.....||...|:...||.....|..

volcanii 916 NFTYTQQAQTDADGEFTMTLPYSTTGYDEYGPDNGYTNVSVRAAGGYAFT 965

maripaludis 853 -------------------------------------------------- 852

volcanii 966 GPTSVTGNSTIVSYQAENVAVDEGLVNGAEDGTVQVTLERNEQELDLPGD 1015

maripaludis 853 --------------------------------------- 852

volcanii 1016 SSSEDSSSEDGTSDGSQTNESASTSTSASVDASAVSAAA 1054
