# Supplementary material for: Complementation of an aglB Mutant of Methanococcus maripaludis with Heterologous Oligosaccharyltransferases
Source: PLoS One. 2016 Dec 1;11(12):e0167611. doi: 10.1371/journal.pone.0167611 (PMC5131992; doi:10.1371/journal.pone.0167611)
Supplement: S5 Fig — (DOCX) [file pone.0167611.s005.docx]

S5 Fig. Alignment of AglB from *Mc. maripaludis* and *S. acidocaldarius*, using EMBOSS Needle.

maripaludis 1 MGEFLNKVSDFFKKNEKIKIILILLFIGMMSFQIRAQTADMAFTDN---- 46

|.|..|.|:.....|.|.

acidocaldariu 1 ----------------------------MQSTSILARIDKFKFLDAIIIG 22

maripaludis 47 -----SYLQDMFSDDNGRMYLTALDPYYYLRMTENYVNNDYSNVGETTVG 91

|.|..:.|.......:...|.:|.. |:.:.....|

acidocaldariu 23 SLALFSILIRIISITAFPQTINGFDSWYLF----------YNALLIVKAG 62

maripaludis 92 IDGENIPYDTIQYAPPG------REAGLVSALSIATVLVYSVW-NSIDST 134

.:...:|.|...:.|.| ...||...:::.:|..||:: .:|..|

acidocaldariu 63 GNWYAVPPDVHAWFPWGYFIELENTIGLPFLVALFSVPFYSIFGQNIVYT 112

maripaludis 135 VTIMNAAFWVPAIMSIFLGIPVFFIVRRNTASNIGGLVGALLLISSPSLL 184

:|::: |.::.....:..|..|...|.|.:||.:.|.:...:|||.

acidocaldariu 113 LTLVS-----PIVLDGIGVVAAFLAVESITNSRVGGYIAAAITAFTPSLT 157

maripaludis 185 YKTSAGFSDTPIFEILPLLFIVWMIMEAIHEQENSKKSGIFGGIAAILIG 234

||...|......:..:.:||.::.:..|| .||..::|..|.|:|.

acidocaldariu 158 YKNILGSLPKTSWGGVFVLFTIYFLSLAI-----KKKKPLYGIPAGIMIF 202

maripaludis 235 LYPMMWSGWWY---AFDITAGFLVLYTAYEYLTKSKNLKNVITTSLITLV 281

|..:.|.|:.| :..|.|..:||: |..:.|:...:|:.

acidocaldariu 203 LANITWGGYTYIDISLAIAAFLIVLF----------NKNDEISAKTLTIS 242

maripaludis 282 G--GAILVSLSTGLSGFINWILSPIGFTVINEATKITGWPNVYMTVSELA 329

| .|.|.|||....||::.:...:...:| .:..:.::|: ...

acidocaldariu 243 GITAAFLTSLSPNTIGFMSEVAHGLALLII----PLFLYLDLYL---RRV 285

maripaludis 330 IPTVTDIIENS---VGNIWLLIAGISGILLSFVSFKHDKQKIDIKYALYL 376

:| .||:::. :|...:|: :|.::|..|:|| .|.|..:|...:

acidocaldariu 286 LP--KDIVDSKNIVIGAAIILL--VSLVVLGSVAFK--VQLIPSRYYAII 329

maripaludis 377 TLWLIATVYAATKGIRFVALMTPALA--IGIGIFAGQIENIIKRY----- 419

..:...||........::.....|:. .|||:|. :||..|

acidocaldariu 330 NPFFQFTVPIDRTVAEYIPQSIAAMIQDFGIGLFL----SIIGIYFLLTR 375

maripaludis 420 EKKVEYILYPVIGILSVITLIKYGGE----LFNILVPTTYVPIAVYLSII 465

::.:..|...|:|..|: ||.. |||. |.|: :.

acidocaldariu 376 KQDMAGIWLVVLGAASI-----YGTSEQPYLFNY---TIYI-------VA 410

maripaludis 466 ALLVLAVYKIIDIISEKEQAVKKVFGILLAFMLVFPSMAAAVPFYTAP-- 513

||..:||.::.....|::..:..:..:.|..:.:......||....||

acidocaldariu 411 ALAGVAVAELFSRFMERKIRIAPILMLTLIGVALLADAGIAVEASYAPQA 460

maripaludis 514 ---------TMNNGWMDSLSWIKSETPENSVVTCWWDNGHIYTWATRKMV 554

|.|..|:.:|.||...||.|:.:..|||.|

acidocaldariu 461 LINSSTSYLTTNYAWISALDWINQNTPNNAFILSWWDYG----------- 499

maripaludis 555 TFDGGSQNTPRAYWVGHAFSTSDENLSVGILRMLATSGDSAYDDDSILIK 604

||: ||. |:....|:

acidocaldariu 500 ------------YWI-HAV------------------GNRTVIDE----- 513

maripaludis 605 KTGSIKDTVDILNKILPLTRTEAKASL-VNNYDLTDAEAEEVLD------ 647

|..|..|:.:..|.: :|| ::.|..||:

acidocaldariu 514 ------------NNTLNGTQIKLMAEMFLNN----ESFAVNVLENDFHLY 547

maripaludis 648 -LTHPKVTNPDYLITYNRMTSIASVWSMFGNWNFSLPASTENSDREMGYY 696

..:|..|.|.|::.|:.:|... |.:.:..|....| |......:||.

acidocaldariu 548 PYGNPNYTRPVYIVAYDAVTEYI-VNNQYPVWFIGYP--TNFPGTFIGYT 594

maripaludis 697 QQLGGSAQDINGTTVV--YIPLQETDSYRVINILEITD-----SEIKSAN 739

..||..|:.|...|.: | .|:|| :|...|.: ::..|..

acidocaldariu 595 TSLGDIAKAIGAMTTIAGY----NTNSY--VNTTYINETASYAAQYNSQL 638

maripaludis 740 AVIDSNNQTSMQSPNFHKLILKVNGNVYEQETNENGDYSEIVRLEKLSDG 789

|.|.:|:.....:|..:..:: |::: |..::.|:.|

acidocaldariu 639 ASIIANSLPMAWTPKTYNSLI---GSMF------------IEAIQSLNQG 673

maripaludis 790 TYQVYAWVSSKNLEDSIYTKLHFLDGYGLEKISLEKESVDPTSYGIQPGF 839

..|....:|...|..|..|.|:..:.. |.:::| .| |

acidocaldariu 674 PVQAPFSISLSQLLQSSSTSLYNPNAL-LPRVNL--------MY-----F 709

maripaludis 840 K-VYSVDYGTDYLN------------------------------- 852

| ||...:.....|

acidocaldariu 710 KPVYIALFPLSVTNALGGEAIVYIMVYIYQFVMPNVIIPPTISTA 754
